# Distinct Modes of Neuritic Growth in Purkinje Neurons at Different Developmental Stages: Axonal Morphogenesis and Cellular Regulatory Mechanisms

**DOI:** 10.1371/journal.pone.0006848

**Published:** 2009-08-31

**Authors:** Annarita de Luca, Stefania Vassallo, Beatriz Benitez-Temino, Gianluca Menichetti, Ferdinando Rossi, Annalisa Buffo

**Affiliations:** 1 Department of Neuroscience, University of Turin, Turin, Italy; 2 Neuroscience Institute of Turin (NIT), Turin, Italy; 3 Rita Levi-Montalcini Center for Brain Repair, National Institute of Neuroscience, Turin, Italy; UMR CNRS 5226 - Université Bordeaux 2, France

## Abstract

**Background:**

During development, neurons modify their axon growth mode switching from an elongating phase, in which the main axon stem reaches the target territory through growth cone-driven extension, to an arborising phase, when the terminal arbour is formed to establish synaptic connections. To investigate the relative contribution of cell-autonomous factors and environmental signals in the control of these distinct axon growth patterns, we examined the neuritogenesis of Purkinje neurons in cerebellar cultures prepared at elongating (embryonic day 17) or arborising (postnatal day zero) stages of Purkinje axon maturation.

**Methodology/Principal Findings:**

When placed *in vitro*, Purkinje cells of both ages undergo an initial phase of neurite elongation followed by the development of terminal ramifications. Nevertheless, elongation of the main axon stem prevails in embryonic Purkinje axons, and many of these neurons are totally unable to form terminal branches. On the contrary, all postnatal neurites switch to arbour growth within a few days in culture and spread extensive terminal trees. Regardless of their elongating or arborising pattern, defined growth features (e.g. growth rate and tree extension) of embryonic Purkinje axons remain distinct from those of postnatal neurites. Thus, Purkinje neurons of different ages are endowed with intrinsic stage-specific competence for neuritic growth. Such competence, however, can be modified by environmental cues. Indeed, while exposure to the postnatal environment stimulates the growth of embryonic axons without modifying their phenotype, contact-mediated signals derived from granule cells specifically induce arborising growth and modulate the dynamics of neuritic elongation.

**Conclusions/Significance:**

Cultured Purkinje cells recapitulate an intrinsically coded neuritogenic program, involving initial navigation of the axon towards the target field and subsequent expansion of the terminal arborisation. The execution of this program is regulated by environmental signals that modify the growth competence of Purkinje cells, so to adapt their endogenous properties to the different phases of neuritic morphogenesis.

## Introduction

The wiring of neural circuits is accomplished during embryonic and postnatal development through a complex multistep process. Neurons first elongate their main axon stem across long distances to reach the appropriate target territory. Here, they develop a highly branched terminal arborization to establish synaptic connections. Elongation of the main axon stem and formation of the terminal arbour represent distinct growth modes [1], which not only occur for each neuronal population at defined developmental stages, but also involve the activation of distinct molecular machineries [2], [3]. Elongating growth is driven by the specialised structure of the growth cone and sustained by the activity of a set of genes, which are mostly downregulated [4], [5] or redistributed to terminal ramifications when the target field is reached [6]. Conversely, the expansion of terminal ramifications is accompanied by the activation of the molecular machinery associated with synaptic assembly and neural transmission [7].

The cellular and molecular mechanisms that mediate the transition from elongating to arborising growth are not well understood. Several lines of evidence indicate that cell-autonomous mechanisms determine the mode of axon growth [8]–[10] and define characteristic neuritic patterns independently of environmental instructive signals [11], [12]. However, extrinsic regulators may also influence neuritogenic processes: distinct target-derived cues can specifically elicit either elongating or arborizing patterns in developing axons [13]–[16], whereas transient environmental signals irreversibly modify the intrinsic growth capabilities of central neurons [17], [18].

To investigate the relative contribution of cell-autonomous factors and environmental cues in the control of distinct axon growth patterns, we examined Purkinje cell (PC) axons. The main PC neurites elongate during embryonic life, when they reach their targets in the cerebellar nuclei [19]–[21]. Later, PC axons develop a conically shaped terminal arbor [22], which expands perinatally to form a highly dense plexus and mature fully functional synapses [23]. Taking advantage of the distinct time-windows in which Purkinje cells carry out elongating or arborizing axonal growth, we examined their neuritic development in dissociated cultures prepared at the relevant ontogenetic stages (respectively embryonic day 17, E17, and postnatal day 0, P0). These cultures reveal that embryonic and postnatal neurons are endowed with intrinsic stage-specific neuritic growth properties that match their neuritogenic pattern *in vivo*. Thus, to ask whether such properties can be modified by extrinsic cues, we co-cultured embryonic PCs with different cellular components of the postnatal cerebellar milieu.

## Results

### Embryonic and postnatal Purkinje cells display distinct axon growth modes

**Figure 1:**
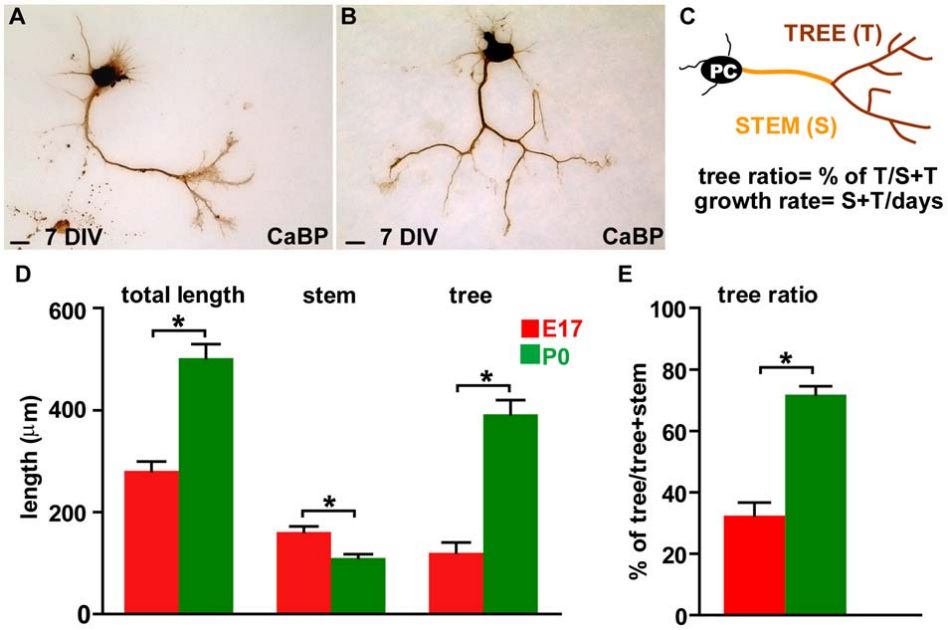
Axon growth pattern of embryonic and postnatal Purkinje cells at 7 days *in vitro*. (A, B) Representative images of calbindin-labeled (CaBP) Purkinje cells (PCs) dissociated at embryonic day 17 (E17) or postnatal day 0 (P0) and cultivated for one week. (C) The cartoon depicts the PC axon compartments considered for the analysis (axon stem, orange, and terminal arbour, brown) and the indexes calculated for morphometric evaluation (tree ratio, growth rate). (D, E) Histograms compare the averages of total length (stem+terminal tree), stem axon length (neuritic segment between the soma and the beginning of the terminal tree), terminal tree (sum of the lengths of segments composing the terminal arbour), and of the tree ratios of cultured E17 and P0 PCs. Asterisks indicate statistically significant differences between E17 and P0 PCs (Mann-Whitney Test, P<0.001). Error bars, SEM. Scale bars, 25 µm.

During embryonic life PCs elongate their main axon stem toward the cerebellar nuclei, while perinatally these neurons develop their terminal arborization. To understand whether PCs engaged in these distinct axonogenic phases share the same competence for axon growth, we dissociated PCs from E17 (elongating phase) or P0 (arborizing phase) cerebella and evaluated *in vitro* their neurite re-extension in conditions of minimal cell-to-cell contact. We first examined neurons that had been maintained for 7 days *in vitro* (DIV). A large fraction of embryonic PCs exhibited an axon stem with a small or absent terminal ramification (Figure 1A), while the majority of postnatal PCs showed a wide and highly branched terminal arbor (Figure 1B). These qualitative observations were further substantiated by quantitative morphometric analyses. The total axon length, calculated by adding up the extension of the main stem and terminal branches (Figure 1C), was significantly higher in postnatal than in embryonic PCs (Figure 1D; E17, 281.40±18.40 µm; P0, 502.57±27.85 µm; Mann-Whitney Rank Sum Test, P<0.001). This difference, however, was not equally distributed among the different axon compartments. The stem axon was longer in E17 neurons (Figure 1D; E17, 161.06±10.92 µm; P0, 110.37±7.13 µm, Mann-Whitney Rank Sum Test, P<0.001), whereas the terminal tree was about three-fold more extended in the P0 population (E17, 120.34±20.27 µm; P0, 392,20±28.29 µm, Mann-Whitney Rank Sum Test, P<0.001). Such different features were further highlighted by calculating the ratio between the tree and the total axon length (Figure 1C; tree ratio = tree/tree+stem): the tree was about 72% of the entire axon in postnatal cells, but only 32% in embryonic neurons (Figure 1E). Altogether, these observations reveal remarkable differences in the modes of axon growth of PCs dissociated at distinct developmental times, suggesting that their competence for neurite elongation varies with age.

To clarify whether embryonic neurons could not develop the terminal arbour due to an unhealthy state, we quantified PC survival. Consistent with previous reports [24], at 7 DIV embryonic PCs were 81.37±11.05% of the initial number of plated cells. On the contrary, at the same time point postnatal neurons were only 25.52±1.51% of the initial value. Therefore, the weak ability of embryonic neurons to develop terminal arbors cannot be related to poor healthy conditions.

**Figure 2:**
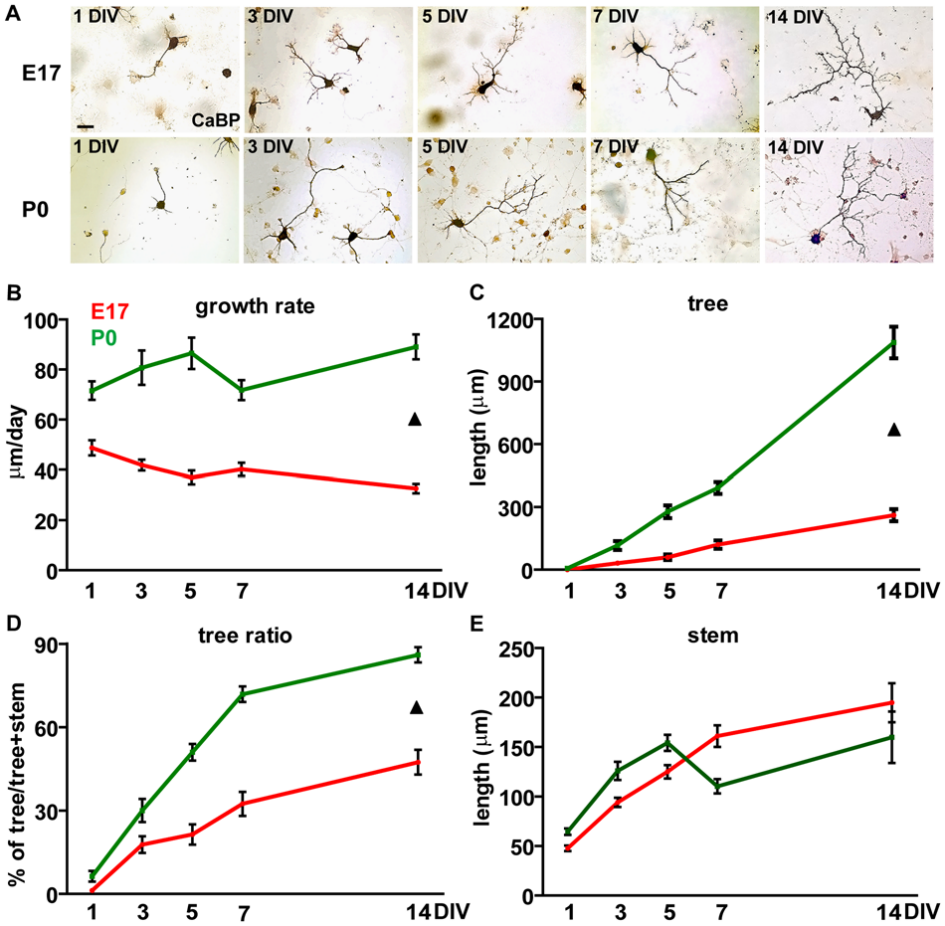
Temporal evolution of axon growth in Purkinje cells plated at different developmental stages. (A) Representative images of calbindin-labeled (CaBP) PC axons at 1, 3, 5, 7 and 14 days *in vitro* (DIV) in embryonic (E17) and postnatal (P0) cultures. Scale bar, 50 µm. (B) Morphometric analysis of E17 and P0 PC axons shows that during the two weeks *in vitro* P0 PCs display a growth rate significantly higher compared to embryonic cells (two way ANOVA, main effect of population, F_1,668_ = 164.22 P<0.001). (C, D) Tree lengths and tree ratios progressively increase in both E17 and P0 PCs, but the P0 values remain constantly higher than those of the embryonic population (two way ANOVA; tree, main effect of population, F_1,668_ = 268.26 P<0.001; tree ratio, main effect of population, F_1,668_ = 110.25 P<0.001). (E) On the contrary, no differences are detected at the statistical level between the two PC populations as far as stem extension (two way ANOVA, P = 0.49). Error bars, SEM. Black triangle: significant main effect of population.

To unveil how the different growth patterns developed over time, the features of PC axons were examined during two weeks after plating (Figure 2A). After one DIV, PCs of both ages already displayed clear outgrowing neurites. Although axonal features were generally similar (Figure 2C–E), P0 PCs already showed a faster growth rate (Figure 1C, 2B; total axon length/days *in vitro*) and this difference, 1.5–2.7 the speed of E17 cells, was maintained during the whole period in culture. Terminal ramifications progressively spread at longer time points (Figure 2A), being always more frequent and extended in postnatal neurons, whose arbors eventually became 4.7 times larger than their embryonic counterparts (Figure 2C). Parallel with arbor enlargement, the tree ratio increased for both populations, being consistently higher for postnatal cells (Figure 2D). On the other hand, PCs of both ages displayed similar absolute lengths of the stem axon (main effect of population F_1,668_ = 0.47, P = 0.49). However, embryonic neurites elongated throughout the whole period, whereas the postnatal ones reached a steady state from 5 days on (Figure 2E). Thus, embryonic and postnatal PCs are endowed with different growth properties, as indicated by distinct growth speed and terminal plexus enlargement.

### E17 and P0 Purkinje cells are heterogeneous and comprise distinct cell classes

**Figure 3:**
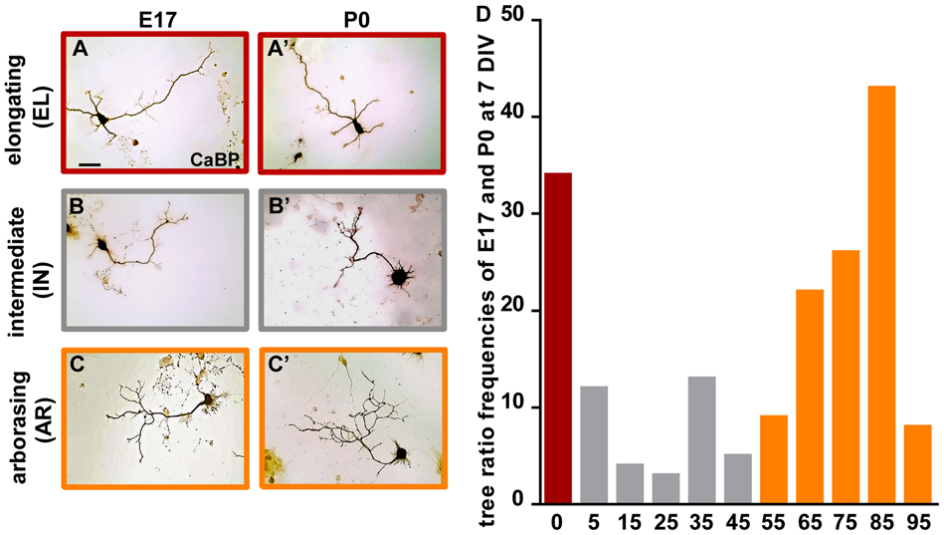
Distinct axon morphologies define three Purkinje axon classes. (A–C') Both embryonic (E17) and postnatal (P0) Purkinje cells (PCs) show a variety of axon morphologies including neurites lacking terminal arbours (A, A'), or carrying small (B, B') or well-developed trees (C, C'). Micrographs show calbindin-positive (CaBP) PCs at 7 days *in vitro* (DIV). Scale bar, 50 µm. (D) The histogram illustrates the frequency distribution of the tree ratios in E17 and P0 PCs (pooled together) at 7DIV. PCs are subdivided in three classes: tree ratio = 0 (brown bar) corresponding to purely elongating axons (EL); tree ratio<50 (gray bars), corresponding to axons in an intermediate pattern of growth (IN); tree ratio>50 (orange bars), corresponding to arborising axons (AR). Labels of the X-axis indicate the central value of each defined class.

The analysis of the growth pattern indicated a prevalence of axon stem extension (elongating growth) for embryonic cells and of terminal plexus enlargement (arborizing growth) for postnatal PCs. Nevertheless, qualitative observations of the cultured cells at both ages revealed a variety of axonal phenotypes (Figure 3A–C′). For instance, while some E17 cells showed a remarkably developed tree and a relatively short stem (Figure 3C), neurons without terminal arbours were present in the postnatal cultures (Figure 3A′). To better characterize such heterogeneity, we took the tree ratio as an index of the predominance of stem or tree compartments in the growth of the axon. By plotting the frequency of different tree ratios of embryonic and postnatal PCs, we first identified two phenotypes: 1) axons with no terminal tree (tree ratio = 0) corresponded to pure elongating growth (brown bars, elongating class, EL, Figure 3A, A′, D); 2) axons that bore a terminal arbour (tree ratio>0), corresponded to the arborizing growth mode and displayed a wide distribution of ratios (gray and orange bars, Figure 3B–C′, D). The latter axons were further subdivided in two classes corresponding to tree ratios higher or lower than 50%. The former include axons whose growth is mostly sustained by arbor expansion (orange bars, arborizing class, AR, Figure 3C, C′, D), whereas the latter represent axons with less expanding arbors, which may be in transition from elongating to arborizing growth (gray bars, intermediate class, IN, Figure 3B, B′, D).

**Figure 4:**
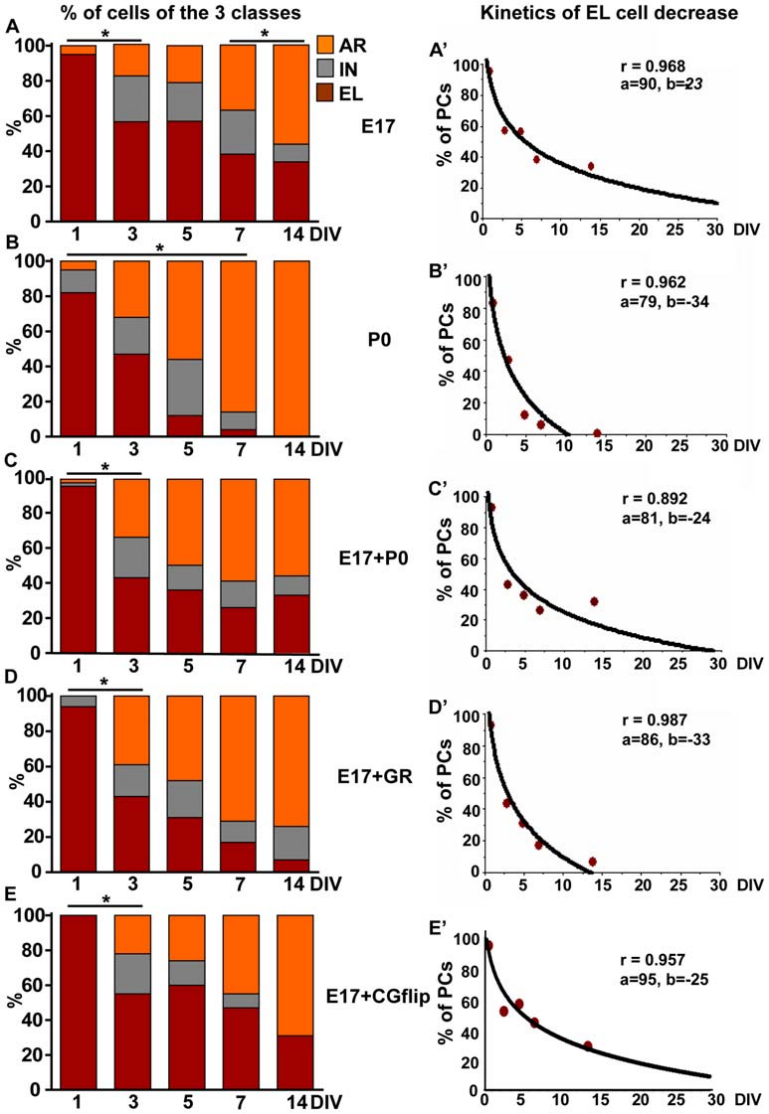
Distribution of Purkinje cell axon classes in different experimental conditions. (A, B) The percentage of embryonic (E17) and postnatal (P0) PCs belonging to the different classes varies over time, leading to a progressive reduction of elongating (EL) cells and to an increase of arborizing (AR) cells. (A) In pure E17 cultures, significant changes occur between 1 and 3 days *in vitro* (DIV; Chi square test, P<0.001) and after 7 DIV (Chi square test, P = 0.03). (B) Postnatal cells vary their distribution until 7 DIV (Chi square test, P<0.001), with no differences between 7 and 14 DIV (Fisher exact test, P = 0.57). Frequency analysis reveals that E17 and P0 PCs significantly differ at 5, 7 (Chi square test, P<0.001) and 14 DIV (Fisher exact test, P<0.001). (C, D) Evolution of E17 axon phenotypes in co-cultures with P0 cells (C, E17+P0) or with granule cells (D, E17+GC). While the postnatal environment modifies the embryonic pattern only at 5 DIV (E17+P0 vs E17, Chi square test, P<0.05), granule cells induce significant changes starting from 3 DIV (E17 vs E17+GR, Chi square or Fisher exact test, P always<0.02). In the E17+P0 cultures, the phenotypic evolution of embryonic axons remains significantly different from that of the postnatal ones (compare C and B E17+P0 vs P0, Chi square or Fisher exact test, P<0.04), whereas GCs trigger a global change toward the postnatal pattern (compare D and B, E17+P0 vs P0, Chi square or Fisher exact test, P>0.05; only at 5DIV P = 0.01). (E) Flipping embryonic PC cultures over GC monolayers to avoid contact-mediated effects (E17+GCflip) does not change the E17 class distribution (Chi square or Fisher exact tests P>0.30). (A–E) Asterisks indicate statistical significant differences between defined time points in each culture condition (P<0.05). (A'–E') Logarithmic fittings [*y = a+bln(x)*] model the decline of the EL PC fraction in the different culturing conditions. IN, intermediate axons.

During the period *in vitro*, all the three classes of axons (EL, AR, IN) were represented in both embryonic and postnatal cultures, but their relative frequencies varied over time (Figure 4A, B). At 1 DIV, both embryonic and postnatal PCs showed a similar distribution (no statistical differences detected, Chi square test χ^2^ *= *5.54, P* = *0.06), with most cells (more than 80%) belonging to the EL class with a short stem and no tree (Figure 3A, A′). At later stages, the fraction of EL axons decreased concomitantly with the development of the terminal arbors (Figure 4A, B). However, both the extent and the kinetics of the transition from elongating to arborizing growth significantly differed in embryonic and postnatal neurons (Figure 4A, B; Chi square test, P<0.001 from 5 DIV on). In embryonic PCs the frequency of EL axons declined slowly and still represented 33.80% of the whole sample at 14 DIV (Figure 4A). On the contrary, in postnatal cultures, EL axons decreased with a faster rate and disappeared completely after two weeks *in vitro*, when all the axons belonged to the AR class (Figure 4B).

The different evolution of the axon growth pattern in E17 or P0 cultures are clearly represented by fitting a logarithmic model (*y = a+bln(x)*) to the percentages of EL axons at different time points (Figure 4A′, B′). The curve obtained for embryonic cells diminished gradually and the estimated percentage of EL cells still remained around 15% at 30 days (Figure 4A′). Conversely, the curve fitted for postnatal cells had a rapid decline and reached the zero value already at 10 days (Figure 4B′). This modelling emphasizes the diverse dynamics of the transition from elongating to arborising growth at different PC maturation stages and, importantly, points to the existence of a fraction of embryonic cells which appear unable to spontaneously develop a terminal arborization.

### Embryonic and postnatal Purkinje axons belonging to the EL or AR class display stage-specific features

**Figure 5:**
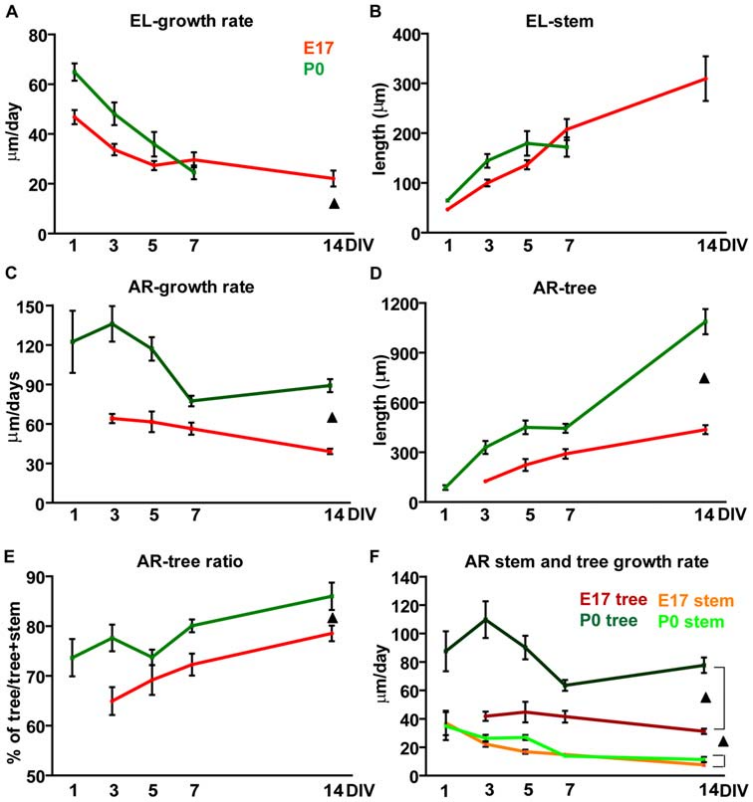
Axonal features and growth of elongating and arborizing Purkinje cells in embryonic or postnatal cultures. (A) The growth rate of both embryonic (E17) and postnatal (P0) elongating (EL) cells progressively decreases during the time *in vitro*, but postnatal axons lengthen faster than their embryonic counterparts during the first week in culture (two way ANOVA, main effect of population P = 0.03). (B) the lengths of stem neurites of both E17 and P0 PCs are similar until 7 DIV (two way ANOVA, F_1,269_ = 2.94, P = 0.09), but during the second week E17 axons grow significantly longer than P0 ones (at 7 DIV, Student's *t* test, P = 0.01). (C–E) Postnatal arborizing (AR) cells extend their neurites faster than E17 PCs (C) and display tree length (D) and tree ratio (E) values consistently higher than embryonic neurons (two way ANOVA, P<0.001). (F) Amongst AR PCs, postnatal arbors expand faster than those of E17 cells (two way ANOVA, F_1,243_ = 80.72, P<0.01). Conversely, the elongation of the stem compartment in AR cells progresses at a similar pace for both E17 and P0 PCs (two way ANOVA, P = 0.09). However, at both ages stem elongation is slower than the enlargement of the terminal tree (two way ANOVA, P<0.001). Missing points in the plot lines are due to the absence of cells the relevant class at specific culturing times. Error bars, SEM. Black triangle: significant main effect of population.

To further characterize the neuritogenic competence of E17 and P0 PCs, we asked whether axons of distinct ages belonging to the same class shared the same growth features. We first compared PCs of the EL class, whose growth is restricted to stem neurite extension. The growth rate of postnatal EL axons was particularly fast just after plating, but decreased considerably during the following days (Figure 5A). On their hand, embryonic EL axons extended according to a slower but more constant pace throughout the whole period (Figure 5A; main effect population F_1,269_ = 4.81 P = 0.03), and eventually developed longer stem neurites than their postnatal counterparts (Figure 5B; Student's *t* test, P<0.05, comparison between E17 PCs at 14 DIV and P0 PCs at 7 DIV).

**Figure 6:**
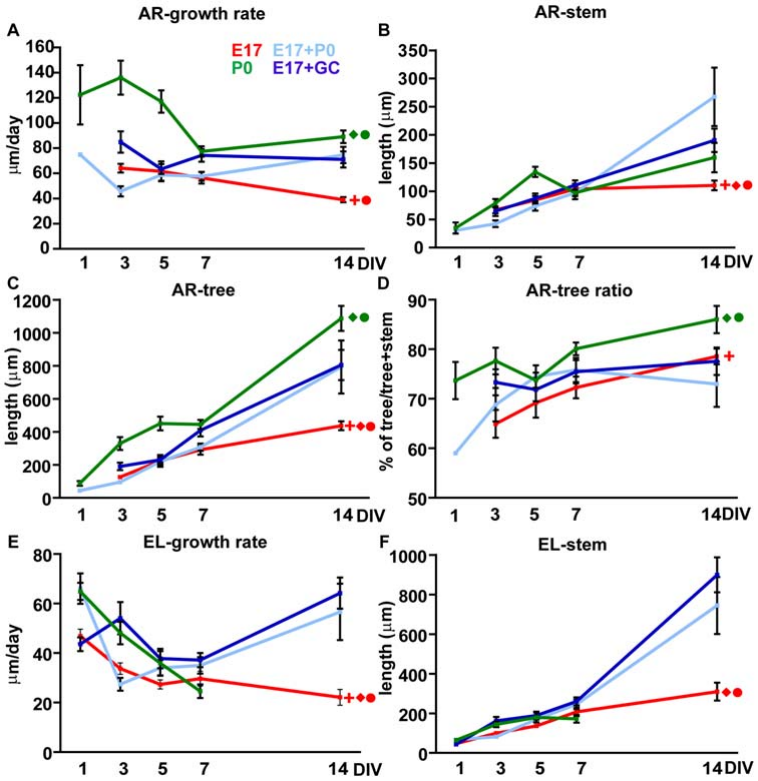
Morphometric parameters of Purkinje axons in pure embryonic (E17) or postnatal (P0) cultures and in co-cultures of embryonic cells with postnatal cells (E17+P0) or with granule cells (E17+GC). (A–D) Parameters of AR axons. (A) P0 and GC environments have a different effect on the growth rate of 17 AR cells (E17+P0: two way ANOVA, P = 0.40; E17+GR: two way ANOVA, P<0.001). However, (B) both culture conditions promote the elongation of the stem compartment (two way ANOVA, always P<0.05) that reaches the value observed in P0 AR cells (two way ANOVA, always P>0.60). (C) Both culture conditions boost the extension of the terminal arbor (two way ANOVA, P≤0.002), although the values are always significantly lower than those of postnatal trees (two way ANOVA, P≤0.002). (D) Because of the concomitant growth of stem and tree compartments (see B and C), the tree ratio is not different from the values of pure E17 cultures (two way ANOVA, P>0.05). (E–F) Parameters of EL axons. (E) Both E17 and P0 environment promote the growth rate of embryonic axons, up to the values typical of their postnatal counterparts (E17+P0 or E17+GC vs E17, two way ANOVA, P<0.001; E17+P0, two way ANOVA: P = 0.66; E17+GC vs P0, two way ANOVA: P = 0.10). (F) In both co-cultures, the length of the stem axon at 14 DIV is significantly longer than that observed in pure E17 cultures (two way ANOVA, P<0.001). Missing points in the plot lines are due to the absence of cells the relevant class at specific culturing times. Error bars, SEM. Significant main effect of population: red cross, E17 vs P0; red diamond, E17 vs E17+P0; red circle, E17 vs E17+GC; green diamond, P0 vs E17+P0; green circle, P0 vs E17+GC.

Within the AR class, postnatal axons were growing faster (Figure 5C; main effect of population F_1, 243_ = 55.67 P<0.001) and extended considerably larger trees (Figure 5D, E; tree length, main effect of population F_1,243_ = 80.72 P<0.001; tree ratio, main effect of population F_1, 243_ = 25.33 P<0.001), whereas their stem axons resulted only slightly longer than embryonic ones (Figure 6B; main effect population F_1,243_ = 8,35 P = 0,004). To further characterize the dynamics of arborizing growth, we analyzed stems and trees separately. In both embryonic and postnatal neurons the high growth speed of AR axons was predominantly due to the fast extension of the terminal tree (Figure 5F). Stem neurites grew about 2–4 times slower than trees (main effect of population F_1, 494_ = 273,83 P<0.001), with no differences between embryonic and postnatal cells (main effect of population F_1,249_ = 2.90 P = 0.09). Furthermore, although the overall growth speed of EL axons of both ages was slower than that of AR axons (Figure 5A; main effect population F_1,546_ = 148.53 P<0.001), the elongation of the stem neurite in AR cells proceeded more slowly than that of EL PCs (compare Figure 5A and F; main effect of population F_1, 504_ = 61.79 P<0.001).

These observations indicate that EL and AR axons of embryonic and postnatal PCs do not share the same growth features. In both classes, embryonic axons grow at a slower pace than postnatal neurites, and EL growth appears consistently slower than AR extension. The high growth rate of AR neurites is mainly sustained by the rapid expansion of the terminal arborization, whereas the stem neurite elongates at a lower speed. Finally, postnatal AR PCs develop larger terminal trees than their embryonic counterparts. At both ages, however, AR neurites extend both the stem and the tree throughout the culturing period, indicating that these axonal compartments grow simultaneously and not sequentially one after the other.

### The growth mode of PC axons is not related to dendritic development

**Figure 7:**
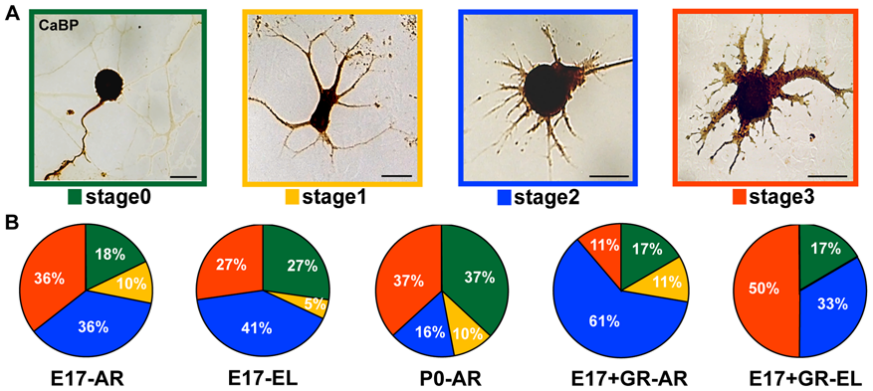
Analysis of Purkinje cell dendritic maturation after two weeks *in vitro*. (A) Representative examples of distinct dendrite maturation stages in calbindin-labeled (CaBP) Purkinje cells (PCs). (B) The pies illustrate the percentages of different dendritic morphologies for EL or AR cells in different culture conditions at 14 days *in vitro*. No significant differences in the distributions are detected between distinct plating ages or PC axon classes (Chi square or Fisher exact tests, P>0.20 for all comparisons). Scale bars, 25 µm.

Previous studies on retinal ganglion neurons indicate that the switch from elongating to arborizing neuritic growth is tightly linked to the initiation of dendritogenic processes [17]. Therefore, we asked whether the EL or AR growth mode of PC axons was related to different stages of PC dendritic maturation, as defined by Baptista et al. [24]. At 14 days *in vitro*, no neurons reached full dendritic maturation (stage 4), but a conspicuous fraction reached stage 3 (27–37%, Figure 7A–B) in both embryonic and postnatal cultures. Despite a tendency for AR cells to bear more mature dendrites, no statistically significant relationships could be established between the dendritogenic stage, the age of the PC or its axon growth mode (Chi square test or Fisher exact tests, P>0.20 for all comparisons). Therefore, the EL or AR elongation pattern of PCs *in vitro* does not appear to be related to the ongoing dendritogenic process.

### The P0 environment stimulates the growth of embryonic Purkinje axons, but does not induce their switch to the arborizing mode

The changes in axon growth ability of PCs at different developmental stages could be the expression of an intrinsic ontogenetic program that progresses regardless of external influences. However, several observations argue against this hypothesis: i) a significant fraction of EL PCs persists in embryonic cultures up to the longest time examined, indicating that they are unable to switch to the AR mode; ii) the growth speed of embryonic and postnatal axons is consistently different throughout the entire period; iii) the axonal features of embryonic and postnatal AR cells are significantly different. These considerations suggest that embryonic PCs do not acquire the growth properties of postnatal neurons by unfolding a purely cell-autonomous program, but require extrinsic instructive information.

**Figure 8:**
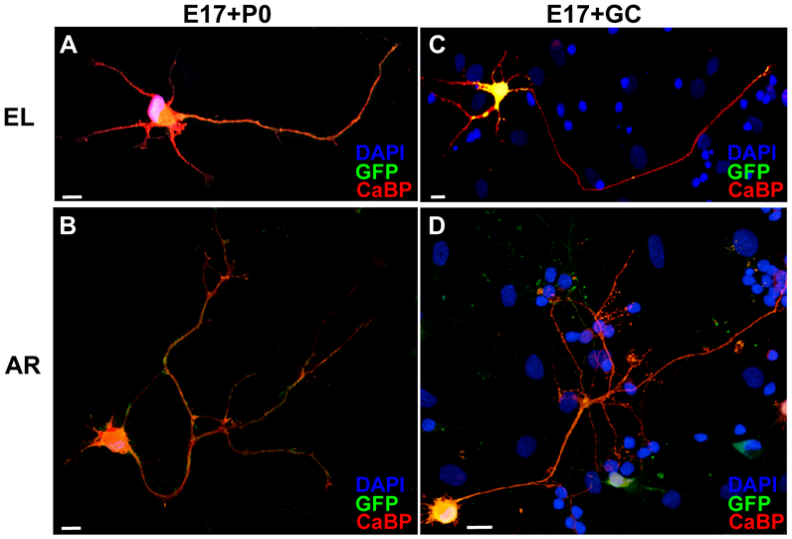
Morphology of embryonic Purkinje cells in the co-cultures. (A–D) Elongating and arborizing phenotypes of calbindin-labeled (CaBP, red staining) embryonic Purkinje cells at 7 days *in vitro* in the postnatal environment *in toto* (A, B, E17+P0) or co-cultivated with granule cell monolayers (C, D, E17+GR). Embryonic cells were distinguished by postnatal cells on the basis of GFP expression (green staining). Co-expression of calbindin and GFP yielded orange staining. At difference with the E17+P0 condition, in E17+GC co-cultures PCs were growing in contact with DAPI-positive cellular elements (blue) (compare A, B vs C, D). Scale bars, 25 µm.

The distinct neuritic growth pattern of embryonic and postnatal neurons could be consequent to the distinct cellular environment of the two cultures [25]. As shown in Table 1, in addition to the different frequencies of PCs, the relative amounts of other types of neurons and of non-neuronal cells were unequal in E17 and P0 cultures (Student's *t* test E17 vs P0 cultures at 7 DIV, P = 0.005, P = 0.015, respectively). Therefore, to ask whether the growth pattern of E17 PC axons could be modified by the postnatal milieu *in toto*, we prepared co-cultures of E17 and P0 cells (P0+E17 co-cultures; 90% P0 cells, 10% E17 cells). In these cultures, postnatal PCs maintained their characteristic pattern of axon growth (not shown), while embryonic neurons (Figure 8A, B) did not display an increased tendency to switch to arborizing growth (compare Figure 4A, B and C; E17+P0 vs E17, Chi square test χ^2^ = 9.59, P<0.05 only at 5 DIV; E17+P0 vs P0, Chi square and Fisher exact test P<0.03 from 5DIV on). Over the examined time, the frequency of embryonic EL cells in the co-cultures decreased following a kinetic similar to that seen in pure embryonic cultures (Fig. 4A′, C′), and still represented about 39% of all PCs at 14 DIV (Figure 4C′).

**Table 1:** Cellular composition of E17 and P0 cerebellar cultures.

culture type	time point	all cells	Purkinje cells	other neurons	non-neuronal cells
**E17**					
	1 DIV	625.00±90.75	123.98±9.95	429.25±66.77	118.75±26.49
	7 DIV	1143.75±110.10	103.42±23.12	250.00±31.38	783.33±92.08
**P0**					
	1 DIV	1027.00±200.50	37.50±7.19	439.50±60.65	539.50±83.79
	7 DIV	1406.67±110.10	11.83±0.83	435.00±49.90	970.00±84.45

The table reports the total number of cells/mm^2^, visualized by DAPI counterstaining, the number of PCs, estimated by counting CaBP-positive cells, of other neurons (CaBP-negative, beta-tubulin-positive cells) and of other non-neuronal DAPI-labeled elements, negative for the tested markers.

Postnatal cues did not alter the overall features of co-cultured E17 AR PCs, which remained clearly distinct from their P0 counterparts (Figure 6A–D; growth rate, tree, tree ratio, E17+P0 vs P0, main effect population always P<0.05; E17+P0 vs E17 main effect population always P>0.05). Nevertheless, although incapable of promoting the transition to the arborizing mode, the postnatal environment stimulated the growth of embryonic neurons. In these cultures embryonic AR PCs eventually developed longer stem axons and larger terminal arbors (see Figure 6A–D, see data at 14 DIV, stem main effect of population F_1,151_ = 9.86 P = 0.002; tree, main effect of population F_1,151_ = 10.40 P = 0.002), while EL PCs showed increased growth speed (Figure 6E; E17+P0 vs E17, main effect population F_1,268_ = 11.90 P<0.001; E17+P0 vs P0, main effect population F_1,203_ = 0.2 P = 0.66,) and stem elongation, reaching values typical of P0 cells (Figure 6F; E17+P0 vs E17 main effect population F_1,268_ = 57.75 P<0.001; E17+P0 vs P0, main effect population F_1,203_ = 0.003 P = 0.95).

### Granule cells stimulate the growth of embryonic Purkinje axons and induce their switch to the arborising mode

We next asked whether exposing E17 cells to enriched components of the postnatal cerebellar environment could induce the transition to the postnatal axon growth pattern. Amongst possible candidates, we tested granule cells (GCs), which are a predominant component of the postnatal cerebellum and well-known regulators of PC maturation [24], [26]. GCs were present in both embryonic and postnatal cultures, although in different amounts (50±8.33 GCs/mm^2^ and 158.33±16.14 GCs/mm^2^ respectively, 261.11±23.98 in E17+P0 cells, 7DIV). To expose embryonic PCs to an environment highly enriched of GCs, E17 cerebellar cells were plated onto GC monolayers. In these cultures, the density of GCs was 808.33±30.05 GCs/mm^2^ (Student's *t* test, P<0.001 for all comparisons with the other culture conditions). Embryonic PCs showed a modest tendency to dendritic maturation (Figure 7B), but displayed a clear-cut switch towards arborizing neuritic growth (Figure 4D). The evolution of PC axon growth pattern during the examined period was consistently different from that of E17 cells alone (compare Figure 4A and D; Chi square test, P always<0.02 from 3 DIV on), and approached the time course seen in P0 cultures (compare Figure 4B and D; Chi square and Fisher exact tests P>0.05 but at 5 DIV χ^2^ = 8.57 P = 0.01). At 14 DIV, EL PCs were only 7% (Figure 4D), while the logarithmic decay curve reached the zero value at about 12 days (Figure 4D′). The different morphometric parameters changed in both EL and AR axons, indicating an overall growth-promoting effect exerted by GCs, which was reflected by the parallel increase of stem length and arbor extension (Figure 6A–F).

Because of the specific culture conditions of this experiment, the embryonic PCs were growing in contact with GCs (Figure 8C, D). In addition, in order to increase GC survival, these cultures were exposed to a high KCl concentration. To ask whether these conditions could be responsible for the changes of PC axon growth pattern, pure E17 cultures were flipped onto GC monolayers. In this experiment, PC axons did not change significantly their growth pattern (Fig. 4E, E′; E17+GCflip vs E17, Chi square or Fisher exact tests always P>0.30), showing that the main effect of GCs on the growth mode of PC axons is exerted through contact-mediated factors, while high KCl concentration has no overt influence. To assess whether the AR growth could be induced by generic contact with any cell type in the cultures, we evaluated the frequency of AR or EL E17 PCs contacted by other cellular elements in E17 or E17+P0 at 14 DIV. As shown in Table 2, in both conditions the relative frequencies of these neuritic phenotypes were similar for isolated PCs or for PCs in contact with other cells (Chi square or Fisher exact tests always P>0.30), indicating a specific effect of GCs in the induction of the AR growth mode.

**Table 2:** Mode of axon growth of isolated and non-isolated PCs.

	E17	E17	E17+P0	E17+P0
	non-isolated PCs	isolated PCs	non-isolated PCs	isolated PCs
EL	33.8%	23.3%	33.3%	20.0%
AR	66.2%	76.7%	66.7%	80.0%

The table reports the frequencies of EL and AR axons in PCs isolated or contacted by other cellular elements in different culturing conditions. The relative frequencies of these neuritic phenotypes were similar for isolated PCs or PCs in contact with other cells (E17, Chi square test χ^2^ =  0.65 P = 0.4; E17+P0, Fisher exact test P = 0.32).

## Discussion

To investigate the mechanisms that regulate the different phases of axonal morphogenesis, we compared the development of E17 and P0 PC axons *in vitro*. Embryonic neurons perform a predominantly elongating growth, carried out at relatively slow speed (40±1.13 µm/day). They can switch to the arborizing mode, but about one third appears totally unable to develop a terminal tree within the examined period. Postnatal PCs grow axons at a higher speed (80±2.59 µm/day) and, within 14 days *in vitro*, all turn to the arborizing phenotype, characterized by the fast expansion of a wide terminal plexus. Embryonic neurites can be induced to spread arbors by GC-derived signals, but their phenotype is not affected by the postnatal milieu *in toto*. On the whole, certain features of PC neurite extension (e.g. growth rate, tree expansion, etc.) appear more related to the neuron age than to the axon growth mode and are scarcely sensitive to culture conditions, indicating that the neuritic pattern expressed *in vitro* is largely determined by intrinsic stage-specific neuronal properties.

### Distinct axon growth modes *in vitro* reflect the ongoing neuritogenic phase *in vivo*

PC axons navigate towards the deep nuclei during embryonic development and develop their terminal trees during early postnatal life [19], [20], [21]. When placed *in vitro*, embryonic neurons mostly extend their stem axon, while postnatal ones soon turn to arbor formation, indicating that they are endowed with distinct age-dependent inclination to carry out specific growth patterns. In agreement with previous observations about dendritic maturation [24], the different ability of E17 or P0 PCs to complete axon development by producing terminal ramifications is not related to their survival *in vitro*, which is much better for embryonic than neonatal neurons. Hence, this different capability cannot be attributed to the health of the cultured neurons. On the other hand, the specific neuritic patterns could be determined by the different environmental conditions provided by embryonic and postnatal cultures. Previous studies on dissociated PCs indicate that exposure to specific substances can stimulate neuritic extension, but do not affect the overall growth pattern [25], [27]. The present co-culture experiments confirm this conclusion and show that switching the axon growth mode requires complex interactions, such as direct cell-to-cell contact with GCs. Therefore, when placed *in vitro* PCs spontaneously reinitiate neuritic development according to a preferential elongating or arborizing pattern that reflects the embryonic or postnatal age when they were dissociated.

### Mechanisms regulating the switch from elongating to arborizing growth of Purkinje cell axons

The diverse axon growth capability of E17 and P0 neurons indicate that during the last days of embryonic development PCs undergo crucial changes for the acquisition of the postnatal arborizing pattern. The existence of embryonic PCs able to develop a terminal arbor *in vitro* suggests that this transition follows an intrinsic developmental clock. Nevertheless, the growth pattern and dynamics of E17 AR axons are overtly different from those of their P0 counterparts, and many embryonic PCs are still unable to form terminal branches at 14 days *in vitro*, long past the age of terminal plexus formation *in vivo*. These observations indicate that the complete transition to the postnatal pattern of axon growth requires specific interactions that are lost when embryonic PCs are isolated from their native environment.

Although E17 PCs are responsive to the P0 environment (their overall outgrowth was stimulated in the E17+P0 co-cultures), the lack of acquisition of the postnatal neuritic pattern indicates that efficient instructors are not acting in the P0 cultures. As potential candidates for this task we tested GCs, whose promoting activity on PC differentiation is well-recognized [20], [28]. GCs represented 4–18% of the entire cell population in E17, P0 and E17+P0 cultures, but exerted a significant effect on PC axon growth only when they were enriched to 60–80% and allowed to take contact with PCs. GCs are presynaptic partners of PCs and their influence on axon development is reminiscent of the regulation exerted by amacrine cells on retinal ganglion neurons [17]. Amacrine cell-derived signals dampen neuritic lengthening and stimulate dendritic extension, leading to the hypothesis that initiation of dendritogenesis restricts the endogenous neuronal inclination for neuritic growth. GCs also regulate the development of PC dendrites [24], [26], [29], [30]. Nevertheless, in our culture conditions we did not detect any clear correlation between the axonal growth mode and the stage of dendritic maturation, indicating that, unlike retinal neurons, dendritogenesis and axonogenesis proceed independently in PCs. Consistent with this conclusion, the manipulation of intrinsic determinants of PC dendritic growth has no obvious effect on their axons [31].

Despite the clear-cut action of GCs on PC axon phenotype, in the pure E17 cultures many PCs spontaneously engage into arbor formation, implying that they had already received instructive information at the time of dissociation. During late embryonic development GC precursors progressively accumulate on the cortical surface to form the external granular layer [20], [28]. Although some interactions with PCs may take place at this stage, in our co-cultures GCs were isolated from P6 cerebella, at a more advanced maturation phase. As a consequence, the interaction between GCs and PCs *in vitro*, albeit functional, is not likely to be the only event responsible for inducing the switch to arbor growth mode. Rather, the peculiar features of embryonic AR neurons indicate that, although E17 PCs are already sensitive to heterochronic cues, acquisition of full-blown arborising growth depends on multiple extrinsic signals and endogenous cellular processes. Indeed, between E17 and P0 PCs undergo several key steps in their maturation, including the arrival of olivary afferents [32], the formation of synapses in the deep nuclei [19], [20] and the establishment of morpho-functional relationships with Bergmann glia [33]. All these factors, plus other ones that could be envisaged, may progressively modify the endogenous properties of maturing PCs and change their competence for sustaining specific patterns of neuritic growth.

### Embryonic and postnatal Purkinje cells show different growth rates

**Figure 9:**
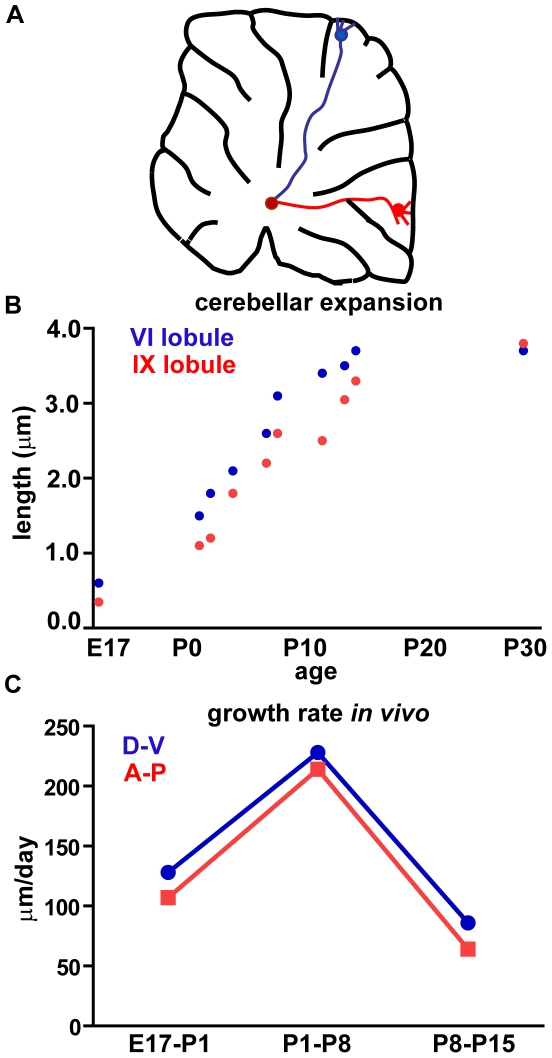
*In vivo* elongation rate of Purkinje cell axons. (A) The cartoon depicts a sagittal section of an adult rat cerebellum, in which PCs (blue in lobule VI and red in lobule IX) send their axons to the deep nuclei region (brown circle). Only Purkinje cells (PCs) localized at the apex of lobules are depicted. (B) These neurons undergo postnatal stem axon elongation to match the corresponding expansion of the cerebellum, which is especially prominent during the first postnatal weeks and declines when the cerebellum reaches its final size. (C) The growth rate of PC axons reaches its maximum during the first postnatal week. Every point represents the average measure obtained from three different rat cerebella. D–V, dorso-ventral; A–P, antero-posterior. These data belong to a previous study [1] and were partly integrated by new measurements and analyses.

Our study shows that postnatal PC axons grow faster than embryonic ones. This feature is different from other CNS neurons, such as retinal ganglion cells [17], and somewhat contradicts the well-established notion that the neuronal ability for neuritogenesis declines as development advances [10]. In the case of PCs, however, growth processes that occur postnatally still involve all the neuritic compartments: terminal ramifications are developed in the deep nuclei and in the cortex, while the stem axon elongates at a considerable pace to match the concomitant volumetric expansion of the cerebellum (Figure 9A–C) [1], [21].

In line with these considerations, AR PC axons *in vitro* undergo simultaneous extension of both stem axon and terminal tree. However, their fast growth rate is mostly sustained by arbor expansion, whereas stem elongation is sluggish and consistently slower than that observed in EL neurites. The latter observation does not match the situation *in vivo*, where stem elongation is slower during embryonic than postnatal life (Figure 9A–C). Embryonic growth of PC stem axons is essentially growth cone-driven pathfinding towards the deep nuclei, whereas postnatal stem elongation is due to interstitial addition [1], stimulated by mechanical tissue stretching [34]. This suggests that the lengthening of stem axons during elongating (embryonic) or arborizing (postnatal) growth is produced by different mechanisms, regulated by distinct stimuli. In the cultures, growth cone-driven elongation is likely not affected, but the mechanical stimulus required to sustain interstitial growth is missing. Accordingly, in both E17 and P0 PCs *in vitro* stem elongation rate is considerably reduced as soon as pure elongating growth is terminated and terminal arbor expansion initiates.

The age-related ability for sustaining interstitial addition rather than growth cone-driven elongation of the stem axon is consistent with the progressive decline of PC axon regeneration during postnatal life [35], [36]. Conversely, the vigorous ability to expand terminal arbours, which steadily increases throughout the period *in vitro*, reflects the inclination of adult PCs for structural plasticity, as observed following axotomy [37]–[40], target denervation [41], removal of extrinsic inhibitory molecules [42], [43], [44], and manipulation of intrinsic determinants [38], [45].

In conclusion, our experiments show that the growth pattern of embryonic or postnatal PCs *in vitro* is determined by the contribution of age-specific properties and mode-associated mechanisms. Regardless of their age, cultured PCs appear to recapitulate an intrinsically coded program, involving initial navigation of the main neurite towards the target field and subsequent expansion of the terminal trees, accompanied by interstitial extension of the stem axon. The execution of this program is regulated by environmental stimuli that modify the growth competence of PCs and modulate the quality and intensity of ongoing processes, so to adapt PC intrinsic properties to the different phases of neuritic morphogenesis.

## Materials and Methods

### Experimental animals

The experiments were performed on Wistar (Harlan, San Pietro al Natisone, Italy) and Green rats, which overexpress the enhanced green fluorescent protein (GFP) under the control of the β-actin promoter (a generous gift from Dr. M. Okabe, Osaka University, Osaka; Japan). From the cerebellum of these animals PCs were dissociated at E17 or P0, whereas P6 rat pups were used for cerebellar granule cultures. All procedures were in accordance with the European Communities Council Directive of 24th November 1986 (86/609/EEC), the NIH guidelines and the Italian law for care and use of experimental animals (DL116/92), and were approved by the Italian Ministry of Health and the Bioethical Committee of the University of Turin. Embryos were obtained by caesarean section from rat dams anesthetized by intraperitoneal injection (i.p.) of ketamine and xilazine (100 mg/kg, Bayer, Leverkusen, Germany and 5 mg/kg, Bayer, respectively). P0 pups were cryoanesthetized in melting ice, while P6 pups were anaesthetised by i.p. injections (see above).

### Cultures of dissociated cerebellar cells

Embryonic and postnatal cerebellar cultures were obtained according to previously established protocols [46]. Culture medium was composed by Eagle's basal medium with Earles's salts (Invitrogen, Gaithersburg, MD) supplemented with glutamine (2 mM, Invitrogen), glucose (32 mM, Sigma), penicillin-streptomycin (20 U/ml, Invitrogen), bovine serum albumin (10 mg/ml, Sigma), an ITS supplement (5 mg/L insulin, 5 mg/L transferring and 5 µg/L selenium, Sigma). The cell suspension was plated on poly-L-lysine-coated (50 µg/ml, Sigma) round glass coverslips (1×10^5^ cells/coverslip, 12 mm diameter, Marienfeld GmbH & Co.KG, Lauda-Konigshofen, Germany). Cells were maintained under controlled conditions (37°C and 5% CO_2_) for up to two weeks, with a medium change after the first week *in vitro*. At 7 days *in vitro* AraC (1 µM, Sigma) was added to P0 cultures to limit the proliferation of glial cells. Cerebellar cells dissociated at E17 from Green embryos were co-cultivated with Wistar P0 cerebellar cells at a 1∶9 ratio (E17+P0; 1×10^4^ E17 cells were mixed to 9×10^4^ P0 cells and plated on the same coverslip). In other sets of experiments, Green E17 cerebellar cell suspensions were co-cultivated with GCs (E17+GC), which were obtained from P6 Wistar pups following pubished protocols [47], [48]. Briefly, the cerebella were dissociated with 0.5% DNAse, the cells obtained were resuspended in culturing medium (see above) added with 25 mM KCl (Sigma) to allow GC survival. GCs were plated to obtain a monolayer (1×10^5^ cells/coverslip) and, after 1 hour at 37°C, 1×10^4^ E17 cells from Green rats were seeded on this layer. In this condition, virtually all PCs had contacts with other cellular components. Therefore, to avoid contact-mediated effects and analyse isolated PCs exposed to GC environment, we carried out additional experiments in which coverslips containing pure E17 cerebellar cultures were flipped onto GC monolayers (E17+GCflip) [24].

### Immunostaining

The cultures were fixed at 1, 3, 5, 7 and 14 days *in vitro* using 4% paraformaldehyde in 0.12 M phosphate buffer, pH 7.4 for 40 minutes and then rinsed 3 times in PBS. For PC identification, cultures were incubated overnight at room temperature with a rabbit polyclonal antibody directed against calbindin (anti-calbindin D28-K, 1∶3000, Swant, Bellinzona, Switzerland), a calcium-binding protein specifically expressed in PCs in the cerebellum [49], diluted in PBS with 0.25% Triton X-100 and 1,5% normal goat serum. Immunohistochemical staining was performed according to the avidin–biotin–peroxidase method (Vectastain ABC Elite kit; Vector Laboratories, Burlingame, CA) and revealed using 3,3-diaminobenzidine (3% in Tris-HCl) as chromogen. To distinguish embryonic PCs in the co-culture experiments, fluorescent double staining was used with rabbit anti-calbindin (1∶1500) and mouse anti-GFP antibodies (1∶700, Invitrogen). The rabbit antibody was revealed by secondary biotinylated antibodies followed by avidin conjugated to Texas Red (1∶200; Invitrogen), and the mouse antibody was labelled by fluorescinated secondary antibodies (1∶200, Vector Laboratories). All cell nuclei were labelled by DAPI (4′, 6 diamidino 2 phenylindole dihydrochloride, Fluka, Buchs, Switzerland, diluted 1∶1000 in PBS). To analyse the cell type composition of the cultures at 1 and 7 DIV, a panel of primary antibodies visualised by immunofluorescence was applied: anti-calbindin (as above) to detect PCs; anti-beta tubulin (1∶100, Sigma) to label all neurons; anti Pax2 (1∶200, Zymed, San Francisco, CA) to reveal young interneurons [50], [51]. GCs were identified according to the following criteria: positivity for beta-tubulin, negativity for PC and interneuron markers, and cell body diameter below 9 µm (see also [29]). The latter value of 9 µm was defined by measuring the size of 100 beta-tubulin labeled neurons that were both Pax-2 and calbindin-negative. Cells positive for DAPI and negative for the tested neuronal markers were considered as non-neuronal phenotypes. Coverslips were mounted using Mowiol (Calbiochem, La Jolla, CA) for microscope visualization and morphological analysis.

### Morphological analysis

Morphometric analysis of PC axons was performed by means of Neurolucida software (MicroBrightField Inc., Colchester, VT) connected to a Nikon E-800 microscope via a color CCD camera. The material was also examined with a Leica TCS SP5 confocal microscope and digital images were adjusted for contrast and assembled with Adobe Photoshop 6.0 (Adobe Systems, Mountain View, CA).

For each experimental condition, a sample of PC, ranging from 40 to 90 cells, with no or minimal contact with other cells, were randomly selected from 3–4 different experiments and their axons carefully reproduced and measured using the Neurolucida software. To define the elongating or arborizing growth mode, morphometric analysis of PC neurites focused on the axon compartments most relevant to distinguish these growth modalities: i) the axon stem, defined as the longest process that originates from the cell body, and ii) the terminal arbor, including all processes emerging from 2 equally thick and symmetric branches budding off at acute angles from the distal end of the axon stem [22] (see Fig 1C). Collaterals budding from the main axon stem at orthogonal angles were not included in the analysis, because they were not relevant to distinguish elongating or arborising patterns.

The degree of maturation of PC dendrites was examined in E17, P0, and GC+E17 cultures at 14 DIV (several tens of Purkinje cells were sampled from each culture condition). Distinct stages of dendrite differentiation were scored following Baptista et al. [24] (stage 0 = no dendrites; stage 1 = multiple thin processes; stage 2 = multiple perisomatic spikes; stage 3 = emerging thick principal dendrite; stage 4 = branched and spiny dendritic tree, see Figure 7), and the corresponding frequencies calculated.

### Quantification of cell survival and cell type composition

E17 and P0 PC density was calculated in 5 quadrants (1 mm^2^ each) localized at central and peripheral sites of the coverslips as defined by two orthogonal coverslip diameters. PCs were identified on the basis of anti-calbindin positivity and only cells extending neurites were considered for survival analysis. For each culture type, PC survival was calculated as percentage of living PCs at 7 DIV over the number of PCs at 1 DIV. With the same sampling method the cell type composition of the cultures was evaluated at 1 and 7 DIV on 3 coverslips from 3 distinct experiments. We counted the total number of cells (DAPI staining), the number of neurons (beta-tubulin staining, also labeled by DAPI), and the number of Purkinje cells (calbindin staining, also labeled by beta-tubulin and DAPI). Analysis of DAPI-positive cells negative for neuronal markers yielded the number of non-neuronal cells. A separate set of coverslips of all experimental sets at 7 DIV was devoted to estimate the number of GCs, identified as described above.

### Statistical analysis

All data are presented as averages±SEM. Statistical evaluation was performed with SigmaStat software package (Jandel Scientific, Germany), applying the Student's *t* test or the Mann-Whitney Rank Sum Test, two way ANOVA followed by the posthoc Bonferroni *t*-test, and Chi-square test or Fisher exact test (when n<5) for frequency analysis. For statistical analysis tree ratio percentages were treated according to the arcsine transformation. In some experimental conditions, EL or AR PCs were not detected at 1 or 14 DIV respectively. In these cases statistical comparisons did not include the missing time points. Effects were considered statistically significant when *P*<0.05. Data fitting was performed with the free Curve Expert 1.3 software.
